# Diaqua­bis(2-bromo­benzoato-κ*O*)bis­(*N*,*N*-diethyl­nicotinamide-κ*N* ^1^)manganese(II)

**DOI:** 10.1107/S160053680901383X

**Published:** 2009-04-18

**Authors:** Tuncer Hökelek, Hakan Dal, Barış Tercan, F. Elif Özbek, Hacali Necefoğlu

**Affiliations:** a Hacettepe University, Department of Physics, 06800 Beytepe, Ankara, Turkey; b Anadolu University, Faculty of Science, Department of Chemistry, 26470 Yenibağlar, Eskişehir, Turkey; c Karabük University, Department of Physics, 78050 Karabük, Turkey; d Kafkas University, Department of Chemistry, 63100 Kars, Turkey

## Abstract

The title Mn^II^ complex, [Mn(C_7_H_4_BrO_2_)_2_(C_10_H_14_N_2_O)_2_(H_2_O)_2_], is centrosymmetric. The mol­ecule contains two 2-bromo­benzoate (BB) and two diethyl­nicotinamide (DENA) ligands and two water mol­ecules, all ligands being monodentate. The four O atoms in the equatorial plane around the Mn atom form a slightly distorted square-planar arrangement, while the distorted octa­hedral coordination is completed by the two N atoms of the DENA ligands in the axial positions. The dihedral angle between the carboxyl group and the adjacent benzene ring is 79.95 (11)°, while the pyridine and benzene rings are oriented at a dihedral angle of 45.66 (6)°. In the crystal structure, inter­molecular O—H⋯O hydrogen bonds link the mol­ecules into infinite chains.

## Related literature

For general background, see: Antolini *et al.* (1982 ▶); Bigoli *et al.* (1972 ▶); Nadzhafov *et al.* (1981 ▶); Shnulin *et al.* (1981 ▶). For related structures, see: Hökelek *et al.* (1995 ▶, 1997 ▶, 2007 ▶, 2008 ▶); Hökelek & Necefoğlu (1996 ▶, 1997 ▶, 2007 ▶).
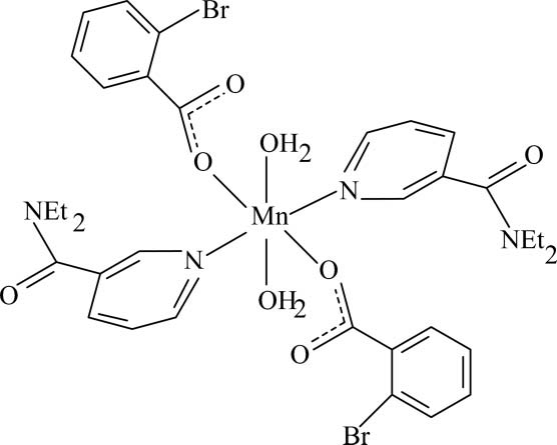


## Experimental

### 

#### Crystal data

[Mn(C_7_H_4_BrO_2_)_2_(C_10_H_14_N_2_O)_2_(H_2_O)_2_]
*M* *_r_* = 847.46
Monoclinic, 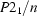

*a* = 13.3022 (2) Å
*b* = 10.2746 (2) Å
*c* = 15.0010 (3) Å
β = 114.798 (1)°
*V* = 1861.21 (6) Å^3^
*Z* = 2
Mo *K*α radiation
μ = 2.56 mm^−1^
*T* = 100 K
0.45 × 0.40 × 0.25 mm

#### Data collection

Bruker Kappa APEXII CCD area-detector diffractometer
Absorption correction: multi-scan (*SADABS*; Bruker, 2005 ▶) *T*_min_ = 0.326, *T*_max_ = 0.525
17076 measured reflections
4637 independent reflections
3922 reflections with *I* > 2σ(*I*)
*R*_int_ = 0.057

#### Refinement

*R*[*F* ^2^ > 2σ(*F* ^2^)] = 0.026
*wR*(*F* ^2^) = 0.071
*S* = 1.03
4637 reflections
233 parameters
2 restraints
H atoms treated by a mixture of independent and constrained refinement
Δρ_max_ = 0.59 e Å^−3^
Δρ_min_ = −0.59 e Å^−3^

### 

Data collection: *APEX2* (Bruker, 2007 ▶); cell refinement: *SAINT* (Bruker, 2007 ▶); data reduction: *SAINT*; program(s) used to solve structure: *SHELXS97* (Sheldrick, 2008 ▶); program(s) used to refine structure: *SHELXL97* (Sheldrick, 2008 ▶); molecular graphics: *ORTEP-3 for Windows* (Farrugia, 1997 ▶); software used to prepare material for publication: *WinGX* (Farrugia, 1999 ▶).

## Supplementary Material

Crystal structure: contains datablocks I, global. DOI: 10.1107/S160053680901383X/xu2511sup1.cif

Structure factors: contains datablocks I. DOI: 10.1107/S160053680901383X/xu2511Isup2.hkl

Additional supplementary materials:  crystallographic information; 3D view; checkCIF report

## Figures and Tables

**Table 1:** Selected bond lengths (Å)

Mn1—O1	2.1238 (10)
Mn1—O4	2.1987 (11)
Mn1—N1	2.3014 (13)

**Table 2:** Hydrogen-bond geometry (Å, °)

*D*—H⋯*A*	*D*—H	H⋯*A*	*D*⋯*A*	*D*—H⋯*A*
O4—H41⋯O3^i^	0.870 (18)	1.86 (2)	2.7207 (16)	168 (2)
O4—H42⋯O2^ii^	0.89 (2)	1.83 (2)	2.6658 (19)	155.1 (19)

Symmetry codes: (i) 
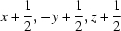
; (ii) 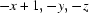.

**Fig. 1.**
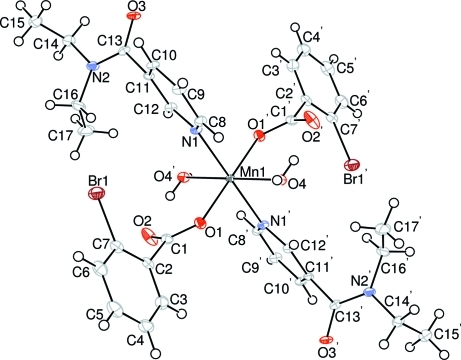
The molecular structure of the title molecule with the atom-numbering scheme. Displacement ellipsoids are drawn at the 50% probability level. Primed atoms are generated by the symmetry operator (1 - x, -y, -z).

## supplementary crystallographic information

### Comment

Transition metal complexes with biochemically active ligands frequently show interesting physical and/or chemical properties, as a result they may find applications in biological systems (Antolini *et al.*, 1982). The structural functions and coordination relationships of the arylcarboxylate ion in transition metal complexes of benzoic acid derivatives change depending on the nature and position of the substituent groups on the benzene ring, the nature of the additional ligand molecule or solvent, and the medium of the synthesis (Nadzhafov *et al.*, 1981; Shnulin *et al.*, 1981). The nicotinic acid derivative *N*,*N*-diethylnicotinamide (DENA) is an important respiratory stimulant (Bigoli *et al.*, 1972).

The structure determination of the title compound, (I), a manganese complex with two 2-bromobenzoate (BB), two diethylnicotinamide (DENA) ligands and two water molecules, was undertaken in order to determine the properties of the ligands and also to compare the results obtained with those reported previously.

Compound (I) is a monomeric complex, with the Mn atom on a centre of symmetry. It contains two BB, two DENA ligands and two water molecules (Fig. 1). All ligands are monodentate. The four O atoms (O1, O4, and the symmetry-related atoms, O1', O4') in the equatorial plane around the Mn atom form a slightly distorted square-planar arrangement, while the slightly distorted octahedral coordination is completed by the two N atoms of the DENA ligands (N1, N1') in the axial positions (Fig. 1).

The near equality of the C1—O1 [1.2611 (17) Å] and C1—O2 [1.2396 (19) Å] bonds in the carboxylate group indicates a delocalized bonding arrangement, rather than localized single and double bonds, and may be compared with the corresponding distances: 1.256 (6) and 1.245 (6) Å in [Mn(DENA)_2_(C_7_H_4_ClO_2_)_2_(H_2_O)_2_], (II) (Hökelek *et al.*, 2008), 1.265 (6) and 1.275 (6) Å in [Mn(C_9_H_10_NO_2_)_2_(H_2_O)_4_].2(H_2_O), (III) (Hökelek & Necefoğlu, 2007), 1.260 (4) and 1.252 (4) Å in [Zn(DENA)_2_(C_7_H_4_FO_2_)_2_(H_2_O)_2_],(IV) (Hökelek *et al.*, 2007), 1.259 (9) and 1.273 (9) Å in Cu_2_(DENA)_2_(C_6_H_5_COO)_4_, (V) (Hökelek *et al.*, 1995), 1.279 (4) and 1.246 (4) Å in [Zn_2_(DENA)_2_(C_7_H_5_O_3_)_4_].2H_2_O, (VI) (Hökelek & Necefoğlu, 1996), 1.251 (6) and 1.254 (7) Å in [Co(DENA)_2_(C_7_H_5_O_3_)_2_(H_2_O)_2_], (VII) (Hökelek & Necefoğlu, 1997) and 1.278 (3) and 1.246 (3) Å in [Cu(DENA)_2_(C_7_H_4_NO_4_)_2_(H_2_O)_2_], (VIII) (Hökelek *et al.*, 1997). In (I), the average Mn—O bond length is 2.1613 (11) Å and the Mn atom is displaced out of the least-squares plane of the carboxylate group (O1/C1/O2) by 0.446 (1) Å. The dihedral angle between the planar carboxylate group and the benzene ring A (C2—C7) is 79.95 (11)°, while that between rings A and B (N1/C8—C12) is 45.66 (6)°.

In the crystal structure, intermolecular O—H···O hydrogen bonds (Table 1) link the molecules into infinite chains, in which they may be effective in the stabilization of the structure.

### Experimental

The title compound was prepared by the reaction of MnSO_4_.H_2_O (0.85 g, 5 mmol) in H_2_O (20 ml) and DENA (1.78 g, 10 mmol) in H_2_O (20 ml) with sodium 2-bromobenzoate (2.23 g, 10 mmol) in H_2_O (50 ml). The mixture was filtered and set aside to crystallize at ambient temperature for 2 d, giving colorless single crystals.

### Refinement

H atoms of water molecule were located in difference Fourier maps and refined isotropically, with restrains of O4—H41 = 0.870 (15) Å and H41—O4—H42 = 106.6 (19)°. The remaining H atoms were positioned geometrically with C—H = 0.93, 0.97 and 0.96 Å, for aromatic, methylene and methyl H atoms and constrained to ride on their parent atoms, with *U*_iso_(H) = *xU*_eq_(C), where *x* = 1.5 for methyl H and *x* = 1.2 for all other H atoms.

### Crystal data

**Table:**

[Mn(C_7_H_4_BrO_2_)_2_(C_10_H_14_N_2_O)_2_(H_2_O)_2_]	*F*(000) = 862
*M**_r_* = 847.46	*D*_x_ = 1.512 Mg m^−^^3^
Monoclinic, *P*2_1_/*n*	Mo *K*α radiation, λ = 0.71073 Å
Hall symbol: -P 2yn	Cell parameters from 9053 reflections
*a* = 13.3022 (2) Å	θ = 2.5–28.3°
*b* = 10.2746 (2) Å	µ = 2.56 mm^−^^1^
*c* = 15.0010 (3) Å	*T* = 100 K
β = 114.798 (1)°	Block, colorless
*V* = 1861.21 (6) Å^3^	0.45 × 0.40 × 0.25 mm
*Z* = 2	

### Data collection

**Table:**

Bruker Kappa APEXII CCD area-detector diffractometer	4637 independent reflections
Radiation source: fine-focus sealed tube	3922 reflections with *I* > 2σ(*I*)
graphite	*R*_int_ = 0.057
φ and ω scans	θ_max_ = 28.3°, θ_min_ = 1.7°
Absorption correction: multi-scan (*SADABS*; Bruker, 2005)	*h* = −17→15
*T*_min_ = 0.326, *T*_max_ = 0.525	*k* = −12→13
17076 measured reflections	*l* = −16→20

### Refinement

**Table:**

Refinement on *F*^2^	Primary atom site location: structure-invariant direct methods
Least-squares matrix: full	Secondary atom site location: difference Fourier map
*R*[*F*^2^ > 2σ(*F*^2^)] = 0.026	Hydrogen site location: inferred from neighbouring sites
*wR*(*F*^2^) = 0.071	H atoms treated by a mixture of independent and constrained refinement
*S* = 1.03	*w* = 1/[σ^2^(*F*_o_^2^) + (0.0399*P*)^2^] where *P* = (*F*_o_^2^ + 2*F*_c_^2^)/3
4637 reflections	(Δ/σ)_max_ < 0.001
233 parameters	Δρ_max_ = 0.59 e Å^−^^3^
2 restraints	Δρ_min_ = −0.59 e Å^−^^3^

### Special details

**Table:**

Geometry. All e.s.d.'s (except the e.s.d. in the dihedral angle between two l.s. planes) are estimated using the full covariance matrix. The cell e.s.d.'s are taken into account individually in the estimation of e.s.d.'s in distances, angles and torsion angles; correlations between e.s.d.'s in cell parameters are only used when they are defined by crystal symmetry. An approximate (isotropic) treatment of cell e.s.d.'s is used for estimating e.s.d.'s involving l.s. planes.
Refinement. Refinement of *F*^2^ against ALL reflections. The weighted *R*-factor *wR* and goodness of fit *S* are based on *F*^2^, conventional *R*-factors *R* are based on *F*, with *F* set to zero for negative *F*^2^. The threshold expression of *F*^2^ > σ(*F*^2^) is used only for calculating *R*-factors(gt) *etc*. and is not relevant to the choice of reflections for refinement. *R*-factors based on *F*^2^ are statistically about twice as large as those based on *F*, and *R*- factors based on ALL data will be even larger.

### Fractional atomic coordinates and isotropic or equivalent isotropic displacement parameters (Å^2^)

**Table:**

	*x*	*y*	*z*	*U*_iso_*/*U*_eq_	
Br1	0.176953 (14)	0.137004 (17)	0.070995 (13)	0.02474 (7)	
Mn1	0.5000	0.0000	0.0000	0.00895 (8)	
O1	0.46956 (9)	0.04297 (11)	0.12498 (8)	0.0153 (2)	
O2	0.35938 (11)	−0.11949 (11)	0.12779 (10)	0.0265 (3)	
O3	0.06295 (9)	0.11431 (10)	−0.39690 (8)	0.0144 (2)	
O4	0.61923 (9)	0.16153 (10)	0.03877 (9)	0.0134 (2)	
H41	0.6063 (18)	0.2388 (16)	0.0553 (16)	0.041 (6)*	
H42	0.632 (2)	0.172 (2)	−0.0145 (17)	0.052 (7)*	
N1	0.35321 (11)	0.13017 (12)	−0.09491 (10)	0.0123 (3)	
N2	0.01375 (11)	−0.00098 (12)	−0.29333 (10)	0.0152 (3)	
C1	0.40415 (13)	−0.01189 (14)	0.15435 (12)	0.0136 (3)	
C2	0.38124 (13)	0.06516 (15)	0.22986 (12)	0.0141 (3)	
C3	0.45806 (14)	0.06857 (16)	0.32723 (13)	0.0200 (4)	
H3	0.5218	0.0181	0.3470	0.024*	
C4	0.44137 (15)	0.14586 (17)	0.39540 (14)	0.0240 (4)	
H4	0.4930	0.1461	0.4606	0.029*	
C5	0.34710 (15)	0.22313 (17)	0.36604 (14)	0.0242 (4)	
H5	0.3369	0.2772	0.4113	0.029*	
C6	0.26873 (15)	0.21982 (16)	0.27000 (13)	0.0219 (4)	
H6	0.2052	0.2706	0.2504	0.026*	
C7	0.28573 (14)	0.14001 (15)	0.20293 (13)	0.0171 (3)	
C8	0.35223 (13)	0.25953 (15)	−0.08357 (12)	0.0139 (3)	
H8	0.4130	0.2986	−0.0338	0.017*	
C9	0.26460 (13)	0.33756 (15)	−0.14278 (12)	0.0149 (3)	
H9	0.2667	0.4270	−0.1325	0.018*	
C10	0.17407 (13)	0.28093 (15)	−0.21728 (11)	0.0134 (3)	
H10	0.1152	0.3315	−0.2591	0.016*	
C11	0.17314 (12)	0.14640 (14)	−0.22823 (11)	0.0113 (3)	
C12	0.26426 (13)	0.07572 (15)	−0.16601 (11)	0.0128 (3)	
H12	0.2636	−0.0141	−0.1740	0.015*	
C13	0.07865 (12)	0.08409 (14)	−0.31173 (11)	0.0117 (3)	
C14	−0.08298 (13)	−0.05404 (16)	−0.37648 (12)	0.0168 (3)	
H14A	−0.0981	−0.1413	−0.3605	0.020*	
H14B	−0.0667	−0.0597	−0.4336	0.020*	
C15	−0.18463 (14)	0.03062 (18)	−0.40047 (13)	0.0235 (4)	
H15A	−0.2464	−0.0065	−0.4547	0.035*	
H15B	−0.1704	0.1165	−0.4176	0.035*	
H15C	−0.2014	0.0354	−0.3442	0.035*	
C16	0.02318 (16)	−0.0346 (2)	−0.19523 (13)	0.0260 (4)	
H16A	−0.0491	−0.0271	−0.1944	0.031*	
H16B	0.0723	0.0271	−0.1480	0.031*	
C17	0.06717 (17)	−0.1714 (2)	−0.16447 (16)	0.0389 (5)	
H17A	0.0675	−0.1908	−0.1018	0.058*	
H17B	0.1412	−0.1772	−0.1598	0.058*	
H17C	0.0206	−0.2325	−0.2124	0.058*	

### Atomic displacement parameters (Å^2^)

**Table:**

	*U*^11^	*U*^22^	*U*^33^	*U*^12^	*U*^13^	*U*^23^
Br1	0.02537 (11)	0.02991 (12)	0.01674 (11)	0.00564 (7)	0.00666 (8)	0.00140 (7)
Mn1	0.01037 (16)	0.00854 (16)	0.00656 (16)	0.00002 (11)	0.00220 (13)	−0.00012 (12)
O1	0.0178 (6)	0.0177 (6)	0.0119 (6)	−0.0049 (4)	0.0078 (5)	−0.0046 (5)
O2	0.0454 (8)	0.0146 (6)	0.0338 (8)	−0.0101 (5)	0.0306 (7)	−0.0087 (5)
O3	0.0192 (6)	0.0130 (5)	0.0066 (6)	−0.0024 (4)	0.0010 (5)	0.0012 (4)
O4	0.0178 (6)	0.0090 (6)	0.0128 (6)	−0.0001 (4)	0.0057 (5)	−0.0003 (5)
N1	0.0136 (6)	0.0121 (7)	0.0091 (7)	0.0002 (5)	0.0027 (5)	−0.0007 (5)
N2	0.0166 (7)	0.0172 (7)	0.0090 (7)	−0.0036 (5)	0.0025 (6)	0.0004 (5)
C1	0.0171 (8)	0.0119 (8)	0.0119 (8)	0.0026 (6)	0.0062 (6)	0.0009 (6)
C2	0.0206 (8)	0.0114 (8)	0.0144 (8)	−0.0035 (6)	0.0115 (7)	−0.0020 (6)
C3	0.0202 (8)	0.0218 (9)	0.0188 (9)	−0.0021 (7)	0.0091 (7)	−0.0042 (7)
C4	0.0269 (10)	0.0301 (10)	0.0152 (9)	−0.0074 (7)	0.0090 (8)	−0.0064 (7)
C5	0.0345 (10)	0.0221 (9)	0.0237 (10)	−0.0058 (7)	0.0198 (9)	−0.0100 (8)
C6	0.0277 (9)	0.0199 (9)	0.0241 (10)	0.0020 (7)	0.0167 (8)	−0.0021 (7)
C7	0.0220 (8)	0.0163 (8)	0.0156 (9)	−0.0026 (6)	0.0105 (7)	−0.0008 (7)
C8	0.0146 (7)	0.0141 (8)	0.0103 (8)	−0.0026 (6)	0.0026 (6)	−0.0029 (6)
C9	0.0192 (8)	0.0099 (7)	0.0134 (8)	0.0001 (6)	0.0046 (7)	−0.0010 (6)
C10	0.0153 (7)	0.0116 (8)	0.0108 (8)	0.0034 (6)	0.0031 (6)	0.0030 (6)
C11	0.0132 (7)	0.0121 (7)	0.0069 (7)	−0.0014 (5)	0.0026 (6)	−0.0001 (6)
C12	0.0159 (7)	0.0100 (7)	0.0107 (8)	−0.0001 (6)	0.0039 (6)	−0.0005 (6)
C13	0.0126 (7)	0.0072 (7)	0.0113 (8)	0.0023 (5)	0.0009 (6)	−0.0006 (6)
C14	0.0180 (8)	0.0154 (8)	0.0133 (8)	−0.0069 (6)	0.0029 (7)	−0.0026 (7)
C15	0.0183 (9)	0.0289 (10)	0.0187 (9)	−0.0024 (7)	0.0034 (7)	0.0039 (8)
C16	0.0259 (9)	0.0396 (11)	0.0107 (9)	−0.0125 (8)	0.0057 (7)	0.0022 (8)
C17	0.0293 (11)	0.0481 (13)	0.0277 (12)	−0.0096 (9)	0.0005 (9)	0.0242 (10)

### Geometric parameters (Å, °)

**Table:**

Br1—C7	1.8999 (18)	C6—H6	0.9300
Mn1—O1	2.1238 (10)	C7—C2	1.392 (2)
Mn1—O1^i^	2.1238 (10)	C7—C6	1.388 (2)
Mn1—O4	2.1987 (11)	C8—C9	1.386 (2)
Mn1—O4^i^	2.1987 (11)	C8—H8	0.9300
Mn1—N1^i^	2.3014 (13)	C9—H9	0.9300
Mn1—N1	2.3014 (13)	C10—C9	1.382 (2)
O1—C1	1.2611 (17)	C10—C11	1.391 (2)
O2—C1	1.2396 (19)	C10—H10	0.9300
O3—C13	1.2444 (18)	C11—C12	1.385 (2)
O4—H41	0.870 (15)	C12—H12	0.9300
O4—H42	0.89 (2)	C13—C11	1.496 (2)
N1—C8	1.3407 (19)	C14—C15	1.519 (2)
N1—C12	1.339 (2)	C14—H14A	0.9700
N2—C13	1.3362 (19)	C14—H14B	0.9700
N2—C14	1.471 (2)	C15—H15A	0.9600
N2—C16	1.465 (2)	C15—H15B	0.9600
C2—C1	1.514 (2)	C15—H15C	0.9600
C2—C3	1.388 (2)	C16—H16A	0.9700
C3—H3	0.9300	C16—H16B	0.9700
C4—C3	1.384 (2)	C17—C16	1.518 (3)
C4—C5	1.391 (3)	C17—H17A	0.9600
C4—H4	0.9300	C17—H17B	0.9600
C5—H5	0.9300	C17—H17C	0.9600
C6—C5	1.380 (3)		
			
O1—Mn1—O1^i^	180.00 (5)	C6—C7—Br1	118.70 (13)
O1—Mn1—O4	89.60 (4)	C6—C7—C2	121.48 (17)
O1^i^—Mn1—O4	90.40 (4)	N1—C8—C9	122.84 (14)
O1—Mn1—O4^i^	90.40 (4)	N1—C8—H8	118.6
O1^i^—Mn1—O4^i^	89.60 (4)	C9—C8—H8	118.6
O1—Mn1—N1^i^	90.07 (4)	C8—C9—H9	120.4
O1^i^—Mn1—N1^i^	89.93 (4)	C10—C9—C8	119.22 (14)
O1—Mn1—N1	89.93 (4)	C10—C9—H9	120.4
O1^i^—Mn1—N1	90.07 (4)	C9—C10—C11	118.39 (14)
O4—Mn1—O4^i^	180.00 (6)	C9—C10—H10	120.8
O4—Mn1—N1^i^	86.81 (4)	C11—C10—H10	120.8
O4^i^—Mn1—N1^i^	93.19 (4)	C10—C11—C13	119.12 (13)
O4—Mn1—N1	93.19 (4)	C12—C11—C10	118.64 (14)
O4^i^—Mn1—N1	86.81 (4)	C12—C11—C13	122.03 (13)
N1^i^—Mn1—N1	180.00 (7)	N1—C12—C11	123.30 (14)
C1—O1—Mn1	129.08 (10)	N1—C12—H12	118.4
Mn1—O4—H41	123.9 (14)	C11—C12—H12	118.4
Mn1—O4—H42	103.8 (16)	O3—C13—N2	122.04 (14)
H41—O4—H42	106.6 (19)	O3—C13—C11	118.24 (13)
C8—N1—Mn1	123.27 (10)	N2—C13—C11	119.71 (13)
C12—N1—Mn1	119.15 (10)	N2—C14—C15	111.35 (14)
C12—N1—C8	117.58 (13)	N2—C14—H14A	109.4
C13—N2—C14	118.59 (13)	N2—C14—H14B	109.4
C13—N2—C16	124.83 (14)	C15—C14—H14A	109.4
C16—N2—C14	116.15 (13)	C15—C14—H14B	109.4
O1—C1—C2	114.36 (13)	H14A—C14—H14B	108.0
O2—C1—O1	126.53 (14)	C14—C15—H15A	109.5
O2—C1—C2	119.11 (13)	C14—C15—H15B	109.5
C3—C2—C1	120.67 (14)	C14—C15—H15C	109.5
C3—C2—C7	118.09 (14)	H15A—C15—H15B	109.5
C7—C2—C1	121.15 (15)	H15A—C15—H15C	109.5
C2—C3—H3	119.5	H15B—C15—H15C	109.5
C4—C3—C2	121.09 (16)	N2—C16—C17	112.52 (16)
C4—C3—H3	119.5	N2—C16—H16A	109.1
C3—C4—C5	119.76 (17)	N2—C16—H16B	109.1
C3—C4—H4	120.1	C17—C16—H16A	109.1
C5—C4—H4	120.1	C17—C16—H16B	109.1
C6—C5—C4	120.18 (16)	H16A—C16—H16B	107.8
C6—C5—H5	119.9	C16—C17—H17A	109.5
C4—C5—H5	119.9	C16—C17—H17B	109.5
C5—C6—C7	119.34 (16)	C16—C17—H17C	109.5
C5—C6—H6	120.3	H17A—C17—H17B	109.5
C7—C6—H6	120.3	H17A—C17—H17C	109.5
C2—C7—Br1	119.80 (12)	H17B—C17—H17C	109.5
			
O1—Mn1—N1—C12	−114.25 (11)	C3—C2—C1—O1	−77.81 (19)
O1^i^—Mn1—N1—C12	65.75 (11)	C3—C2—C1—O2	101.89 (19)
O4—Mn1—N1—C12	156.15 (11)	C7—C2—C1—O1	98.69 (18)
O4^i^—Mn1—N1—C12	−23.85 (11)	C7—C2—C1—O2	−81.6 (2)
O1—Mn1—N1—C8	66.53 (12)	C1—C2—C3—C4	175.46 (14)
O1^i^—Mn1—N1—C8	−113.47 (12)	C7—C2—C3—C4	−1.1 (2)
O4—Mn1—N1—C8	−23.07 (12)	C5—C4—C3—C2	−1.0 (2)
O4^i^—Mn1—N1—C8	156.93 (12)	C3—C4—C5—C6	2.0 (3)
O4—Mn1—O1—C1	179.20 (13)	C7—C6—C5—C4	−0.8 (2)
O4^i^—Mn1—O1—C1	−0.80 (13)	Br1—C7—C2—C1	4.3 (2)
N1^i^—Mn1—O1—C1	−94.00 (13)	Br1—C7—C2—C3	−179.13 (11)
N1—Mn1—O1—C1	86.00 (13)	C6—C7—C2—C1	−174.25 (14)
Mn1—O1—C1—O2	15.7 (2)	C6—C7—C2—C3	2.3 (2)
Mn1—O1—C1—C2	−164.62 (10)	Br1—C7—C6—C5	−179.91 (12)
Mn1—N1—C8—C9	178.27 (11)	C2—C7—C6—C5	−1.4 (2)
C12—N1—C8—C9	−1.0 (2)	N1—C8—C9—C10	−0.3 (2)
Mn1—N1—C12—C11	−178.48 (11)	C11—C10—C9—C8	1.8 (2)
C8—N1—C12—C11	0.8 (2)	C10—C11—C12—N1	0.7 (2)
C14—N2—C13—O3	−3.8 (2)	C13—C11—C12—N1	175.36 (13)
C14—N2—C13—C11	175.90 (13)	C9—C10—C11—C12	−1.9 (2)
C16—N2—C13—O3	−175.90 (15)	C9—C10—C11—C13	−176.78 (13)
C16—N2—C13—C11	3.8 (2)	O3—C13—C11—C10	61.46 (19)
C13—N2—C14—C15	−89.33 (17)	O3—C13—C11—C12	−113.21 (16)
C13—N2—C16—C17	−109.22 (18)	N2—C13—C11—C10	−118.22 (16)
C14—N2—C16—C17	78.48 (18)	N2—C13—C11—C12	67.11 (19)
C16—N2—C14—C15	83.48 (18)		

Symmetry codes: (i) −*x*+1, −*y*, −*z*.

### Hydrogen-bond geometry (Å, °)

**Table:**

*D*—H···*A*	*D*—H	H···*A*	*D*···*A*	*D*—H···*A*
O4—H41···O3^ii^	0.87 (2)	1.86 (2)	2.7207 (16)	168 (2)
O4—H42···O2^i^	0.89 (2)	1.83 (2)	2.6658 (19)	155 (2)

Symmetry codes: (ii) *x*+1/2, −*y*+1/2, *z*+1/2; (i) −*x*+1, −*y*, −*z*.
